# Acoustic voice characteristics with and without wearing a facemask

**DOI:** 10.1038/s41598-021-85130-8

**Published:** 2021-03-11

**Authors:** Duy Duong Nguyen, Patricia McCabe, Donna Thomas, Alison Purcell, Maree Doble, Daniel Novakovic, Antonia Chacon, Catherine Madill

**Affiliations:** Voice Research Laboratory, Faculty of Medicine and Health, D18, Susan Wakil Health Building, Camperdown Campus, The University of Sydney, Western Avenue, Sydney, NSW 2006 Australia

**Keywords:** Disease prevention, Health services

## Abstract

Facemasks are essential for healthcare workers but characteristics of the voice whilst wearing this personal protective equipment are not well understood. In the present study, we compared acoustic voice measures in recordings of sixteen adults producing standardised vocal tasks with and without wearing either a surgical mask or a KN95 mask. Data were analysed for mean spectral levels at 0–1 kHz and 1–8 kHz regions, an energy ratio between 0–1 and 1–8 kHz (LH1000), harmonics-to-noise ratio (HNR), smoothed cepstral peak prominence (CPPS), and vocal intensity. In connected speech there was significant attenuation of mean spectral level at 1–8 kHz region and there was no significant change in this measure at 0–1 kHz. Mean spectral levels of vowel did not change significantly in mask-wearing conditions. LH1000 for connected speech significantly increased whilst wearing either a surgical mask or KN95 mask but no significant change in this measure was found for vowel. HNR was higher in the mask-wearing conditions than the no-mask condition. CPPS and vocal intensity did not change in mask-wearing conditions. These findings implied an attenuation effects of wearing these types of masks on the voice spectra with surgical mask showing less impact than the KN95.

## Introduction

Facemasks are an essential piece of personal protective equipment (PPE) and can be broadly categorized into respirators, medical masks (including surgical masks and procedure masks), and woven fabric (cloth) masks^1^. Respirators and surgical masks provide different levels of barrier to prevent infectious transmission via aerosols and droplets^2^. Masks with higher barrier levels (e.g. N95) are used in aerosol generating procedures (AGPs) and other high risk activities^1^. During non-aerosol generating protocols, surgical masks offer a similar degree of protection to N95 masks against viral respiratory infections including coronaviruses in health care workers (HCWs)^3^. Although surgical masks do not provide the same level of protection as N95 masks, they prevent some aerosols and droplets from being released from phonation and respiratory activities, contributing to reducing the risk of transmission^4^. In the SARS-CoV2 pandemic (COVID-19), such masks have been recommended for use by not only HCWs but also the general public in areas with known or suspected widespread transmission, high population density, or settings where physical distancing cannot be effectively achieved^5^. Although masks are effective PPE^4^, wearing a mask negatively affects the physiological and psychological performance of HCWs^6^.

Masks also interfere with effective verbal communication. Certain masks particularly the N95 respirators can impact speech understanding by listeners^7^. Word intelligibility dropped between 1 and 17% while wearing respirators commonly used by HCWs in which N95 mask resulted in a mean (standard deviation, SD) of modified rhyme test (MRT) score of 83 (16.2)% compared to 92 (5.8)% in non-mask controls^8^. The use of N95 mask in background noise resulted in a significant decrease in speech perception accuracy^9^. Speaking while wearing a mask at longer distances decreases speech perception accuracy by an even greater magnitude than not wearing a mask^10^. A mask also physically creates a visual barrier precluding lip reading^11^, precluding communication cues in people with hearing loss and communication disabilities such as aphasia^12^. From a user’s perspective, wearing masks increased perception of vocal effort, reduced auditory feedback, and difficult coordination of speech and breathing^13^. Understanding the aspects of the voice changes whilst wearing a mask is important so clinical decision, making and choice of mask is appropriate to meet infection control and optimal verbal communication.

Although it is believed that facemasks attenuate sound transmission like a low-pass filter^10,14^, little information is available on voice characteristics whilst wearing a facemask. The scarce literature on the topic suggests possible changes in the speech spectrum. Mendel et al.^15^ compared speech spectral levels calculated as total root mean square (RMS) power from the Connected Speech Test (CST) stimuli produced by one speaker with and without wearing a surgical mask. They found a significant difference in the total RMS power between the two conditions. However, the affected frequency band was not reported. Atcherson et al.^11^ found that the total RMS values of speech signals from the CST stimuli were significantly higher when not wearing a mask compared to the conditions with a mask. They also did not mention which frequency range was affected by the mask. Goldin et al.^14^ utilised a head and torso simulator to play white noise via the model’s mouth without a mask and with a surgical mask and a N95 respirator. They found that facemasks attenuated the sound levels at frequency regions between 2 and 7 kHz by 3–4 dB for the surgical mask and nearly 12 dB for the N95 mask compared with the non-mask condition. Their model lacked natural speech features while its face contour and surface were not similar to human face contour and skin, affecting fitting levels of the masks. However, based on their findings it seems reasonable to hypothesize that mask wearing would attenuate speech spectra at similar frequency bands.

Clear and natural speech production is necessary in accurate speech understanding and requires less listening effort than degraded speech^16^. Given the widespread use of facemasks in COVID-19 pandemic, it seems reasonable to further clarify the characteristics of the voice signal in speech of vocally healthy speakers who are wearing a mask. Given the above-mentioned findings of the modification of the speech spectra by the mask, the present study quantified the low- and high-frequency energy regions. Spectral analyses not only give information about the overall spectral shape that may be meaningful in speech perception^17^ but also provide important acoustic correlates of voice quality^18^. These spectral measures were selected as both the low and high frequency regions also contribute to speech recognition^19,20^. Low-frequency spectral bands are important in recognizing vowels^21,22^ and voiced fricative consonants^23^. High frequency spectral energy makes a significant contribution to speech recognition^24,25^ including the recognition of vowels^26^, voiceless and voiced fricative consonants^27^, spoken and sung text^28^, and speech recognition in noise^29^. It has also been shown that the high-frequency region provided perceptual cues for speaker identity^30^ and gender identification^31^. In addition, the high frequency region plays an important role in the perception of clear speech: a shift of energy concentration toward higher frequency regions contributes to the clear speech effect for normal-hearing listeners^32^.

Presumably, the quality and audibility of the voice might also change whilst wearing a facemask as previous studies have observed voice changes in phonation with the mouth covered^33^. This change may interfere with auditory-perceptual voice judgment by speech language pathologists (SLPs) and ear nose and throat specialists (ENTs). Dysphonic voice quality has also been proven to result in reduced comprehension of speech content by listeners^34,35^. Wearing a mask may add to this effect by increasing the difficulty of understanding speech of an individual with dysphonic voice. Both voice quality and audibility can be effectively examined using acoustic analysis, which is a non-invasive objective assessment. Traditional acoustic measures of voice quality are based on frequency-based measurements^36^ and include fundamental frequency (F0)^37^ and noise (harmonic-to-noise ratio, HNR)^38–41^. Amongst these, HNR has been used as a measure of vocal clarity^42^. The vocal signal can also be analysed based on spectral-based measurement of vowel and connected speech, which does not depend upon reliable tracking of vocal F0^43^. The cepstral peak prominence (CPP) has been shown to have stronger weighted correlations with overall voice quality than other acoustic measures^44^. Given that it is a measure of periodicity and harmonics strength, a signal with a strong harmonic structure would have a higher CPP than aperiodic signals^45^. It has been considered a significant predictor of dysphonic severity^46^. However, there are inherent limitations of cepstral analysis, that is, it is affected by vowel types and vocal intensity^47^, vocal tasks^48^, vocal tract^49^, and the algorithm of software packages^48,50^. Vocal audibility can be examined both by spectral energy at different frequency bands and sound intensity, which can also be estimated from the acoustic signals.

During the COVID-19 pandemic two types of masks were commercially available in Australia, including standard surgical mask and KN95 mask (China GB2626-2006)^51^. KN95 mask provided similar protection characteristics to N95 mask^52^. The major filtering and fitting characteristics of the KN95 mask as provided by 3 M^52^ were as follows: Filter performance ≥ 95%; Flow rate = 85 L/min; Inhalation resistance ≤ 350 Pa; Exhalation resistance ≤ 250 Pa; and Total inward leakage < 8%. The total inward leakage indicates the amount of an aerosol that enters the mask via both filter penetration and face-seal leakage^52^. Presumably, the higher barrier level a mask can provide, the greater impact it would have on the voice signals. The aims of the present study were to (1) examine the acoustic characteristics of voice and speech whilst wearing either a surgical mask or a KN95 mask; and (2) compare the acoustic measures between the standard surgical mask and KN95 mask. We hypothesized that: (1) Low- and high-frequency spectral levels, HNR, CPPS, and vocal intensity would change during wearing these facemasks; and (2) Changes in these acoustic measures would be more pronounced with KN95 mask than with standard surgical mask.

## Methods

### Ethical approval

**Figure 1:**
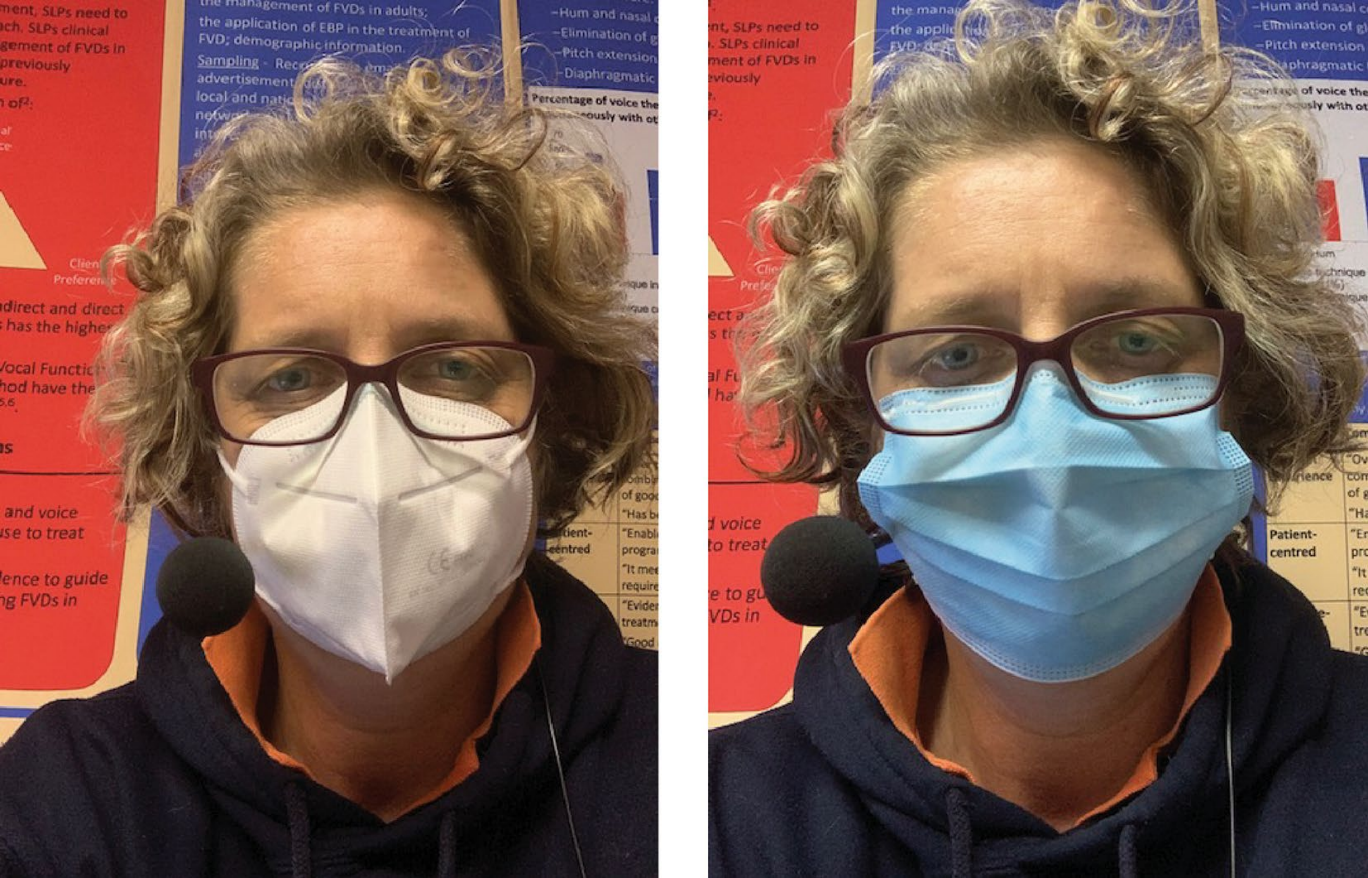
Voice recording with a KN95 (left) and surgical mask (right).

The voice and speech data analysed in this study was part of a larger project which was approved by the Human Research Ethics Committee of The University of Sydney (protocol number: 2020/399). Informed consent was obtained from all participants to participate in this study. Informed consent to publish was also obtained from a participant for publication of identifying information/image (Fig. 1) in an online open-access publication. The present study was implemented in accordance with relevant ethical guidelines and regulations. The measurement procedures used in this study conformed to the standards set by the latest revision of the Declaration of Helsinki.

### Participants

Sixteen participants took part in this study (12 females, 4 males) with mean age = 43 years (range = 24–61). All were English speakers, non-smokers, and did not report any voice nor hearing problems at the time of the study. Participants were otolaryngologists (n = 2), practicing speech language pathologists (n = 13), and a registered nurse working in an Ear Nose and Throat clinic (n = 1).

### Voice recordings

Due to social distancing measures during the COVID-19 pandemic, it was impossible for participants to have their voices recorded in the same recording environment. Therefore, voice recordings took place in a room at the practicing clinic of the participants with ambient noise ranging from 33.3 decibels (dBA) to 58.0 dBA. Participants were required to use their habitual voice to read the following standardised tasks: three repetitions of the sustained vowel /a/ for at least 10 s, the Consensus Auditory-Perceptual Evaluation of Voice (CAPE-V) phrases^53^, and the Rainbow Passage^54^. These tasks were produced in three conditions with the speaker (1) not wearing a mask, (2) wearing a surgical mask, and (3) wearing a KN95 mask (Fig. 1). The order of conditions was randomised across speakers to minimize biases related to intra-speaker variability in phonation and potential compensation whilst wearing a mask. When wearing these masks, participants were required to use the highest level of fitting to ensure maximal barrier level. They were required to press the nose metal bar so that it fit tightly to the nose contour. The straps of the mask were securely placed behind the auricles and the lower side of the mask was pulled fully downward so that it covered the chin completely (Fig. 1). It has been known that in unfavourable/challenging speaking conditions, speakers may adapt a phonation style that helps improve clear phonation^55,56^. Therefore, we required participants to maintain similar habitual voice in terms of pitch, loudness, and phonation type throughout recording sessions both with and without a mask to minimise intra-speaker variability in voice production.

All voice signals were captured using an AKG C520 ear-mounted microphone^57^ placed at a constant distance of 6 cm, 45° off the mouth axis and were analog-to-digital converted using a professional external sound card (Roland Quadcapture^58^) at 44.1 kHz and 16-bit resolution. The signals were processed and saved to a laptop computer using the Audacity sound editing software in *.wav format^59^. Calibration of sound level in the voice signals was deemed unnecessary given that the data were used to test within-subject effects of mask and non-mask conditions.

### Acoustic analysis

Voice samples were edited in Audacity to extract the middle 3 s of the sustained /a/ vowel, the 3rd CAPE-V phrase (CAPEV-3), and the 2nd and 3rd sentences of the Rainbow Passage (RP23). All acoustic data were measured using Praat version 6.0.39^60^.

#### Mean spectral level in low (0–1 kHz) and high (1–8 kHz) frequency ranges

Spectral levels in the 0–1000 Hz and 1000–8000 Hz were measured in Praat for the /a/ vowel (averaged from three repeats) and RP23. 1000 Hz was the cut-off between the low- and high-frequency regions in this study as the spectral region above 1000 Hz has been frequently used in investigating the role of different spectral regions in speech perception^25^. Consonant noise is mainly concentrated at frequency regions above this frequency^61^. Further, the 1000 Hz cut-off has been used in studies involving spectral characteristics of voice quality^62–64^. The upper limit of 8 kHz was used as extended high frequency ranges above this frequency have minor value in speech perception^65^. The protocols in Praat were as follows: From Analyse spectrum =  > To LTAS, set bandwidth = 100 Hz and click OK. From Query =  > Get mean, then frequency bands were set with averaging method being “dB”. The output was then copied to an Excel spreadsheet for analysis.

#### Low/high spectral energy ratio between 0–1 and 1–8 kHz (LH1000)

We also evaluated the low/high energy ratio (reflecting spectral slope) which is a ratio of spectral energy levels between the low and high frequency ranges to investigate how this would be affected given the impact of mask-wearing on the speech spectrum. The low/high ratio using a 1000 Hz cut-off value (LH1000) has been used frequently in voice and speech research and has been shown to reflect voice quality^62,63^, vocal load^64^, sentence prominence in speech^66^, and the effects of language^67^.

The low/high energy ratio between spectral areas below 1 Hz and between 1–8 kHz was measured for the /a/ vowel (averaged from three repeats) and RP23 using the long-term average spectra (LTAS) function in Praat. The command to obtain this measure in Praat was as follows: From Analyse spectrum =  > To LTAS, set bandwidth = 100 Hz and click OK. From Query =  > Get slope, set averaging method = dB, low band = 0–1000 Hz, high band = 1000–8000 Hz and click OK. The value that Praat software provided was measured in dB.

#### Harmonics-to-noise ratio

Praat (version 6.0.39) was also used to measure harmonics-to-noise ratio (HNR) from the sustained /a/ vowel. The 3-s vowel sample was open and highlighted in Praat editing window from which HNR was obtained using the command Voice report within the Pulses menu. Data was averaged from three repeats. Prior to measurement of HNR, all edited vowel samples were signal-typed by the first author (D.D.N.) and a research assistant using criteria recommended by Titze^68^ and Sprecher et al.^69^ This was conducted using narrow-band spectrograms generated in Praat using settings described in Sprecher et al.^69^. Signal typing was performed visually by comparing each spectrogram picture with the exemplar signal types. Signal typing was deemed necessary because the measurement of HNR relies on reliable estimation of F0, which is only feasible in type 1 and type 2 signals^69^.

#### Cepstral peak prominence smoothed

The voice cepstrum is obtained by a Fourier transform of the logarithm power spectrum^70^. A cepstral peak is identified within the dominant ‘rahmonic’ corresponding to the fundamental period from which CPP is calculated as the amplitude between the peak and the regression line directly below it^45^. Smoothing the individual cepstra before extracting the cepstral peak and calculating CPP can improve prediction accuracy^18^. CPP-smoothed (CPPS) was measured in Praat using settings as follows^71,72^: Pitch floor (Hz) = 60, Time steps (s) = 0.002, Maximum frequency (Hz) = 5000, Pre-emphasis from (Hz) = 50, Time averaging window (s) = 0.01, Quefrency averaging window (s) = 0.001, Peak search pitch range (Hz) = 60–330, Tolerance (0–1) = 0.05, Interpolation = Parabolic, Subtract tilt before smoothing = No, Tilt line quefrency range (s) = 0.001–0.0 (= end), Line type = Straight, Fit method = Robust.

#### Vocal intensity

Vocal intensity (dB) was also measured from the vowel, the 3rd CAPEV phrase, and the 2nd and 3rd sentences of the Rainbow Passage using Praat with default settings. Intensity values were not calibrated to real sound pressure level as the purpose of the study was to examine within-speaker effects.

### Quality check of voice recordings and reliability analysis

Because voice recordings took place in different clinic rooms with different levels of background noise, audio files were examined for signal-to-noise ratio (SNR) using a Praat script called Speech-to-noise ratio /Voice-to-noise ratio v.01.01^73^. Only samples with a SNR greater than 30 dB were used for acoustic analyses^74^.

The sound files of four participants in all conditions [n = 4 × 3 conditions (no-mask, surgical mask, KN95) = 12], were randomly selected and analysed a second time by a research assistant for HNR and LH1000 to calculate inter-rater reliability using Intraclass Correlation Coefficient (ICC, two-way mixed, consistency type). The results are shown in Table 1, which indicate excellent reliability. ICC was 1.00 for LH1000 as the measurement of this was fully automated using edited voice samples. The slightly lower ICC values for HNR resulted from possible differences between the raters in selecting (highlighting) the vowel segment for HNR measurement in Praat editing window.

**Table 1:** Inter-rater reliability of acoustic analyses.

Measures	ICC (*p*)	
	Single measures	Average measures
HNR	0.985 (0.000)	0.992 (0.000)
LH1000 vowel	1.000 (0.000)	1.000 (0.000)
LH1000 of RP23	1.000 (0.000)	1.000 (0.000)

RP23 = The 2nd and 3rd sentences of the Rainbow Passage.

### Statistical analyses

Data were managed in Microsoft Excel 365^75^ and analysed using IBM SPSS Statistics v.25.0^76^ and Prism v8.1.2^77^ for Windows. One-way repeated-measures analysis of variance (ANOVA) was used to examine the effects across three conditions (no-mask, surgical mask, and KN95 mask) on acoustic measures. Significant main effects were evaluated with Bonferroni-adjusted tests. Prior to analyses, normal distribution of the data was examined using Kolmogorov–Smirnov tests^78^. Mauchly’s test of sphericity was performed before ANOVA and, if sphericity assumptions were not met, a Greenhouse–Geisser adjustment was used. Effect size was calculated using partial Eta squared (η^2^). Effect sizes of 0.01, 0.1, and 0.25 indicated small, medium, and large effects, respectively^79^. Where normality assumption was not met, the Friedman test was used to compare data across non-mask, surgical mask, and KN95 conditions. A significance level of 0.05 was used.

## Results

### Mean spectral levels at low and high frequency regions

#### Mean spectral levels in 0–1 kHz region

**Figure 2:**
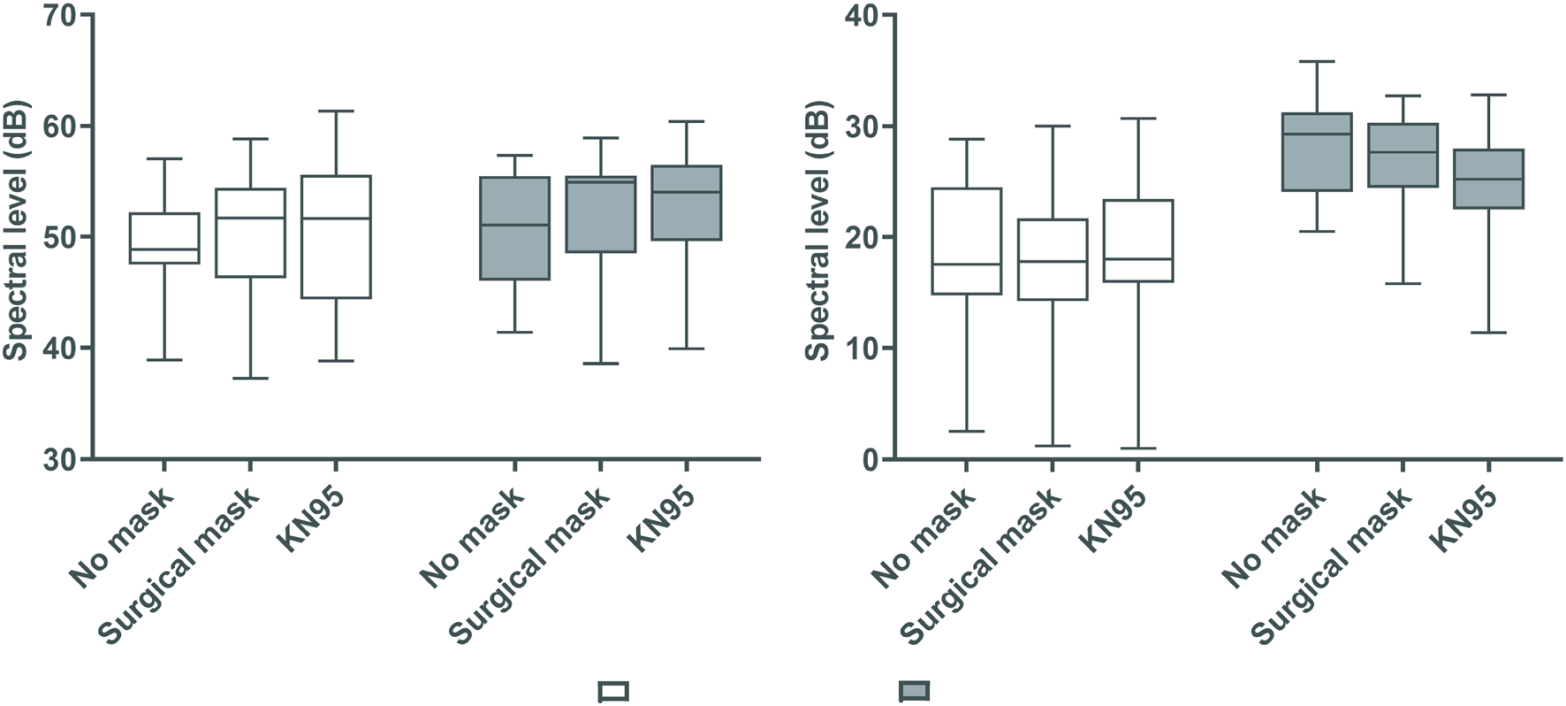
Mean spectral levels at low and high frequency regions across conditions. As sound pressure level was not calibrated, spectral levels were normalized so that the lowest value equalled zero. RP23 = The 2nd and 3rd sentences of the Rainbow Passage.

Figure 2 showed mean spectral levels at both frequency bands 0–1 kHz and 1–8 kHz. This figure shows that this spectral measure did not change across conditions for both vowel and connected speech. For sustained /a/ vowel phonation, no significant main effects of mask-wearing were found: F(2, 22) = 0.396, *p* = 0.678, partial η^2^ = 0.035. For RP23, there was also no significant main effects of masks in the 0–1 kHz range F(1.235, 13.588) = 0.808, *p* = 0.410, partial η^2^ = 0.068.

#### Mean spectral levels in 1–8 kHz region

Figure 2 shows mean spectral levels for both vowel and connected speech in the 1–8 kHz region. For vowel production, no significant main effects of wearing a facemask were observed F(2, 22) = 0.024, *p* = 0.963, partial η^2^ = 0.002. However, for RP23, wearing a facemask affected mean spectral levels in the 1–8 kHz region: There was a significant main effect of mask-wearing on this measure F(1.173, 11.735) = 16.951, *p* = 0.001, partial η^2^ = 0.629. Post-hoc tests showed that, compared with the non-mask condition, the KN95 mask attenuated the spectral levels in the 1–8 kHz region by 5.2 dB (*p* = 0.005) while the surgical mask attenuated the spectral levels in this region by 2.0 dB (*p* = 0.014).

### Low/high spectral ratio (LH1000)

**Figure 3:**
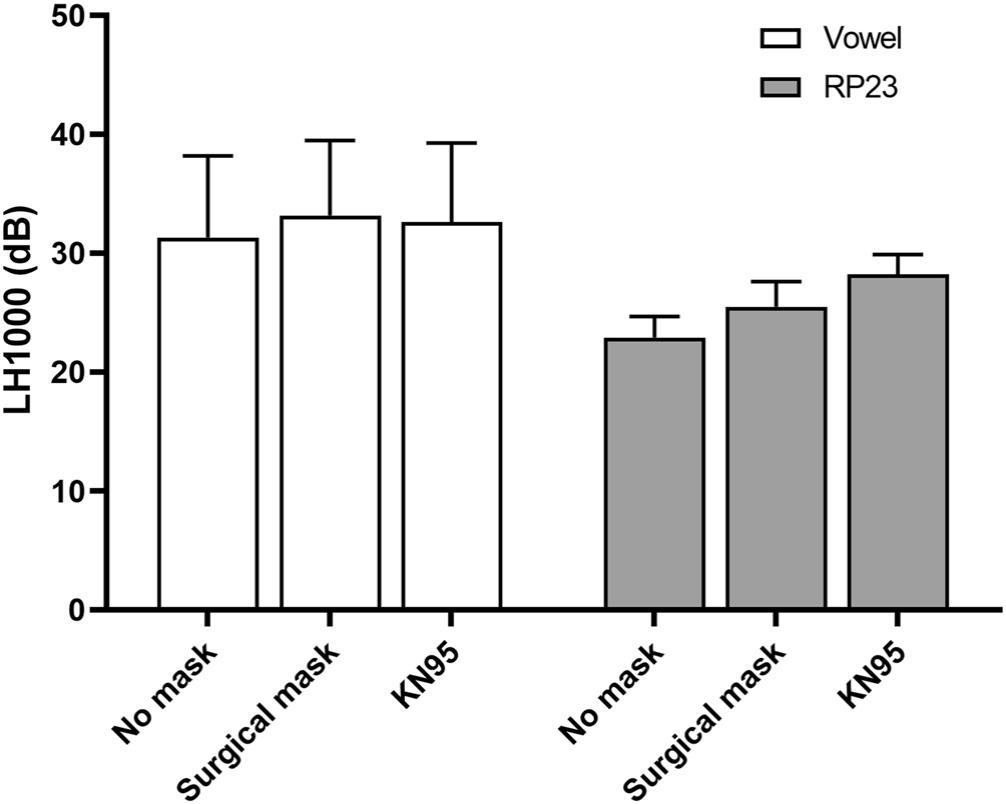
Mean low/high ratio at 1000 Hz for all conditions. Error bars indicate standard deviation. RP23 = The 2nd and 3rd sentences of the Rainbow Passage.

LH1000 was calculated for the /a/ vowel and RP23. Figure 3 shows mean and SD of LH1000 for both tasks. One-way repeated-measures ANOVA was calculated to compare data across non-mask, surgical mask, and KN95 mask. For the sustained vowel, no significant main effects were observed: F(2, 22) = 0.949, *p* = 0.402, partial η^2^ = 0.079. In Fig. 3, LH1000 for the vowel produced did not change significantly across non-mask and mask conditions.

For RP23, a significant main effect was present: F(1.279, 14.073) = 84.346, *p* = 0.000, partial η^2^ = 0.885. Figure 3 shows that LH1000 of RP23 was the lowest for the non-mask [mean (SD) = 23.0 (1.7) dB], higher for the surgical mask [mean (SD) = 25.5 (2.2) dB], and highest for the KN95 mask condition [mean (SD) = 28.2 (1.7) dB]. Pairwise Bonferroni-adjusted comparisons showed that wearing a KN95 mask increased the LH1000 of RP23 by 5.2 dB (*p* = 0.000) and wearing a surgical mask increased the LH1000 of RP by almost 2.5 dB (*p* = 0.000).

### Harmonics-to-noise ratio

**Figure 4:**
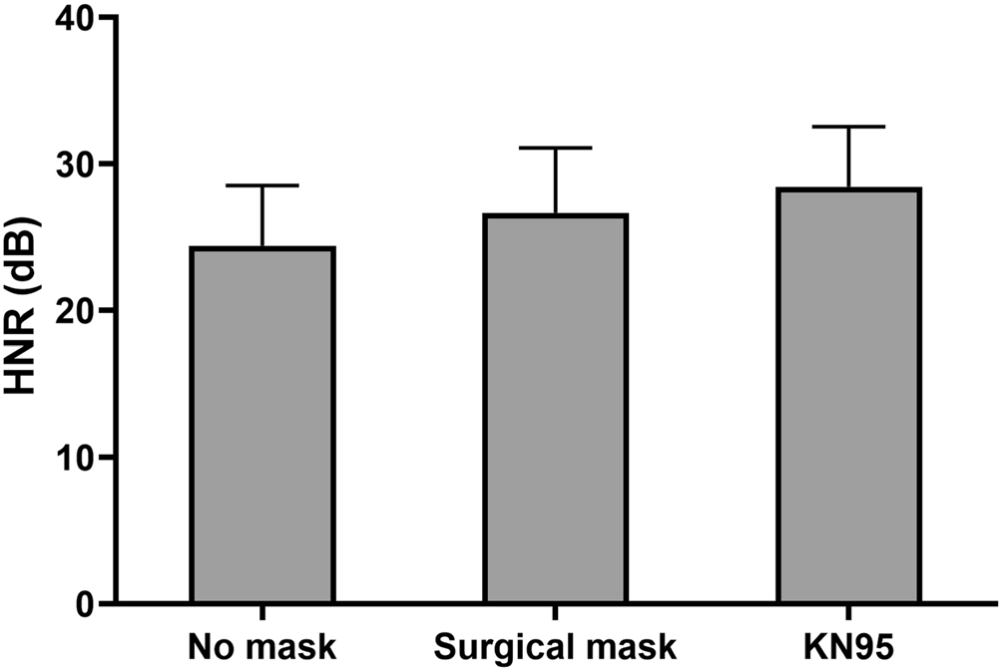
Harmonics-to-noise ratio in all conditions. Error bars = standard deviation.

Figure 4 shows HNR in all experimental conditions. HNR was compared across non-mask, surgical mask, and KN95 mask conditions using one-way repeated-measures ANOVA; significant main effects were found: F(2, 22) = 14.749, *p* = 0.000, partial η^2^ = 0.573. Post-hoc Bonferroni-adjusted tests showed that HNR significantly increased with wearing either a surgical mask or KN95 mask. In the non-mask condition, the mean (SD) of HNR was 25.0 (3.5) dB. HNR values increased significantly to 27.3 (4.5) dB (*p* = 0.004) and 28.4 (4.1) dB (*p* = 0.000) when wearing a surgical mask and KN95 mask, respectively.

### Cepstral peak prominence smoothed

**Figure 5:**
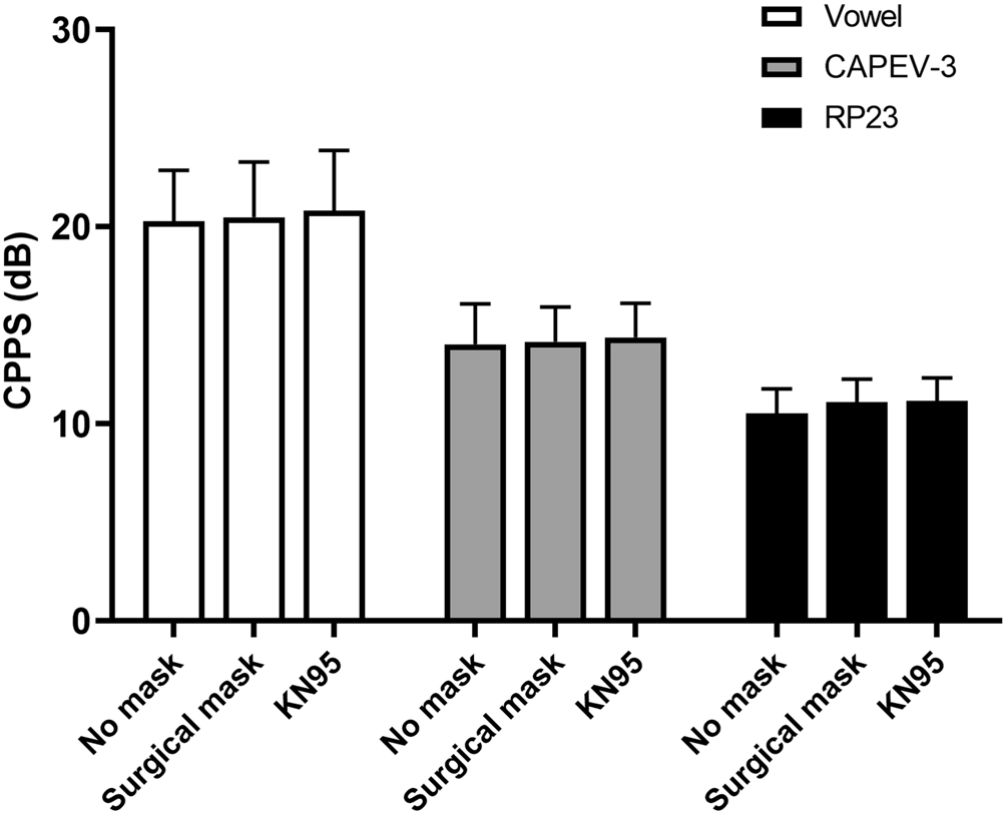
Cepstral peak prominence smoothed (CPPS) for all conditions. CAPEV-3 = The third CAPE-V phrase. RP23 = The 2nd and 3rd sentences of the Rainbow Passage.

Figure 5 shows that CPPS did not change across all conditions for all three vocal tasks. One-way repeated-measures ANOVA comparing no-mask, surgical mask, and KN95 mask showed no statistically significant effects of mask-wearing on CPPS of vowel [F(2, 22) = 0.695, *p* = 0.51, partial η^2^ = 0.059], CAPEV-3 [F(1.326, 13.260) = 0.013, *p* = 0.954, partial η^2^ = 0.001], nor RP23 [F(1.218, 13.393) = 4.313, *p* = 0.051, partial η^2^ = 0.282].

### Vocal intensity

The intensity of the vowel increased slightly in both mask conditions however the changes were not statistically significant (Table 2). Similarly, there were no main effects of mask-wearing on vocal intensity of the CAPEV-3 and RP23.

**Table 2:** Mean (SD) of vocal intensity (dB) in each condition and *p* values from repeated-measures ANOVA.

Tasks	Non-mask	Surgical mask	KN95	*p*
Vowel	64.0 (7.0)	65.8 (7.2)	66.7 (7.5)	0.188
CAPEV-3	63.9 (4.9)	64.5 (5.7)	64.8 (6.1)	0.595
RP23	59.7 (5.0)	61.3 (5.2)	60.9 (5.1)	0.290

CAPEV-3, The third CAPEV phrase; RP23, The 2nd and 3rd sentences of the Rainbow Passage.

Data from all three conditions were used to calculate the Pearson’s correlation coefficient (r) between vocal intensity and CPPS. There were moderate correlations between vocal intensity and CPPS (Vowel: r = 0.595, *p* = 0.000; CAPEV-3: r = 0.522, *p* = 0.000; and RP23: r = 0.366, *p* = 0.014).

## Discussion

In this study we hypothesized that voice quality measures (e.g. spectral levels at low- and high-frequency, HNR and CPPS) and audibility measure (vocal intensity) would change during wearing either a surgical mask or KN95 mask. The data confirmed our hypotheses for spectral characteristics, showing a significant decrease in mean spectral levels at high frequency regions (1–8 kHz) and an increase in LH1000 (implying steeper spectral slope) when wearing either a surgical mask or KN95 mask. HNR improved significantly in both mask-wearing conditions. However, CPPS and vocal intensity did not change.

The decreased spectral levels in the 1–8 kHz region was in agreement with a previous modelling experiment. In Goldin et al.’s study^14^, sound levels between 2 and 7 kHz was attenuated by 3–4 dB with the surgical mask and by approximately 12 dB with the N95 compared with the non-mask condition. In the present study we found that the spectral levels at the 1–8 kHz region was attenuated by 2.0 dB by surgical mask and 5.2 dB by KN95 mask. The findings may be explained as degrading/attenuating effects of mask on spectral levels. These appeared to depend upon the filter performance and the level of fitting of the masks in which the KN95 mask outperforms standard surgical masks. Although both our and Goldin et al.’s studies found greater impact of masks with higher barrier levels (N95/KN95), levels of fitting may have varied in Goldin et al.’s study as they used a model in which face contour and skin characteristics were not similar to human subjects. Perceptual studies have shown that masks with higher filtering characteristics impact more on speech i.e. respirators decreased speech intelligibility scores while surgical masks did not show significant differences in speech intelligibility compared with no-mask condition^8^. Mendel et al.^15^ also found that surgical masks did not have a negative effect on speech understanding in both normal hearing and hearing loss groups. Taken together, these data showed that KN95 masks differed from standard surgical masks in level of degradation of the signals, hence listener’s mechanisms of adaptation in speech perception might be different when listening to speech produced whilst wearing these masks. This would imply that surgical masks seem to be a more appropriate choice over the KN95 (similarly to N95 masks in specifications^52^), given that there is insufficient evidence for selecting N95 masks over surgical masks for protecting HCWs against infectious disease transmission^80^.

The spectral energy in this study was calculated using data combined from both genders because of the within-subject study design. Between-speaker and between-gender variability in the level of changes in spectral characteristics as a result of the mask was therefore not examined. It is well known that speech spectra carry information of both the larynx (voice source) and the vocal tract (filter)^81^ and reflect gender characteristics^31^. Apart from common spectral features across speakers^82^, there may also be variabilities in characteristics of the speech spectrum across speakers. Between-speaker variability has been observed in source spectral shape and spectral noise in both genders, F3 and F4 and formant dispersion in female voices; and spectral slope in the higher frequencies (from the fourth harmonic to the harmonic closest to 2 kHz and from the harmonic closest to 2 kHz to the harmonic closest to 5 kHz) for male voices^82^. These are within the 1–8 kHz range investigation in the present study. As such, the impact of mask on phonation may not be the same for all speakers. For these reasons spectral measures should be analysed separately for each gender. However, the limited sample size (12 females and 4 males) did not allow sufficient statistical power for each group. Testing spectral measures that characterize between-speaker variability can help evaluate the impact more specifically for each gender. Speaker-specific and gender-specific source-filter characteristics and phonation and articulation strategy with and without wearing a mask were therefore not examined. It is not known whether male or female voices were affected similarly by wearing a mask.

It is possible that the speakers in this study used an individual strategy to adapt their phonation style, which may also account for the findings. Although the participants were instructed to keep their phonation style constant, it was impossible to control for this. Adaptation in mask-wearing condition may include unconsciously increasing vocal projection to compensate for presence of the mask. Increased HNR has been observed in speakers with increased vocal intensity^83^. However, in the present study vocal intensity was not significantly different across the three conditions. It was therefore not possible to confirm whether the improved HNR resulted from vocal adaptation. In addition, why HNR was higher in mask-wearing conditions whilst CPPS remained unchanged was unclear. Although HNR and CPPS actually reflect different phenomena in voice quality^49^, the non-significant changes in CPPS and vocal intensity across conditions did not appear to support the compensation assumption. In addition, phonation compensation in mask wearing may include increased vocal effort, as reported in a previous study^13^. Increased vocal effort seems associated with a different trend of spectral change. In vocally healthy speakers McKenna and Stepp^84^ observed that typical phonation style had the highest L/H ratio (a ratio of low to high spectral energy with cut-off at 4 kHz) and this measure decreased steadily from mild to maximal effort (i.e. decreased spectral slope in vocal effort). Meanwhile, the two mask-wearing conditions in the present study showed an opposite trend in spectral slope as expressed in the LH1000 findings.

Regardless of the actual mechanisms, the findings implied that several important high-frequency components might change during wearing these masks. These include the higher formants that are necessary to identify linguistic contents of segmental units. Previous studies have shown that high frequency energy is important to recognize vowels^26^. As the second formant for the vast majority of English vowels exist above 1 kHz^85^, it is likely that correct identification of vowels will be compromised if frequencies above 1 kHz are attenuated by the wearing of a facemask. The recognition of voiceless consonants (e.g. /s/ and /f/) also requires detection of high frequency regions above 1 kHz^61^. For example, the noise component produced in /s/ is centred between 5 and 6 kHz^86^. The changes in higher frequency regions also imply that the quality of the speech signal will be affected as the frequencies of voiceless plosives, fricatives and affricates occur within 2–4 kHz, as does place of articulation^86^. Although the findings were not specific to any specific segmental acoustic measures of speech recognition, accurate recognition of speech may be compromised when the speaker is wearing a mask, as demonstrated in some previous studies^8,9^. It is important to note that the data in this study was recorded in sound-proofed or quiet conditions, while in reality, people are wearing masks in noisy environments, which will most likely further reduce the audibility of the speech signal. The degradation of speech spectral signals, associated with background noise and with acoustically reflective surfaces (e.g. hard uncarpeted floors) will further reduce audibility of the signal and speech perception, making it difficult for listeners, especially those with hearing loss, to perceive and understand the speech signal in such high noise level environments. Further research using landmarks for speech recognition^87^ would help clarify the specific impact of wearing a mask on important linguistic content e.g. vowels and consonants.

The improved HNR in mask-wearing condition agreed with previous findings that people with voice disorders sound less dysphonic when their mouth is covered. For example, hand-over-mouth has been believed to reduce vocal constriction and increase frontal/oral resonance, facilitating more effective voice production^88^. Wearing a semi-occluded ventilation mask (SOVM)^89^ in people with normal voices and functional dysphonia also showed significant differences between SOVM and control in aerodynamic, electroglottographic, and acoustic measures. The improvement in HNR can also be explained using similar mechanisms as for spectral findings i.e. filtering out/attenuation of glottal noise from the signals by the masks. Given that glottal noise components in voice typically appear in high frequency range^90,91^, noise attenuation by the masks may make spectral energy at low frequency more dominant relative to high frequency, hence improving HNR. In addition, the masks might affect vocal tract resonance, leading to changes in nasal-oral coupling with more nasal resonance involved because of increased impedance at the mouth opening whilst wearing the masks. A previous study has shown that when phonation changed from vowel to nasal, HNR increased by 1.5 dB^49^.

We did not observe significant changes in vocal intensity for all vocal tasks in mask conditions. This agreed with our non-significant findings on CPPS and was contrary to our expectation that wearing a mask would attenuate intensity. This is possibly due to the calculation of intensity by the Praat program, as default settings of intensity are calculated on frequency settings. In this study, intensity calculations were conducted on frequencies between 75 and 500 Hz. Additionally, although the participants were asked to maintain approximately similar vocal intensity across conditions, they might inadvertently increase their volume slightly in mask conditions to compensate for the altered feedback created by the mask. In addition, the intensity values were obtained from the signals recorded using a cardioid (directional) microphone at distance of 6 cm from the mouth. Whether this applied to perception/audibility of loudness/vocal intensity at real communication distances is not known.

Several issues need consideration in future studies. The main limitation of this study was that it did not evaluate speech intelligibility as the vocal tasks used were standardized for assessing voice quality and not for speech intelligibility. Future studies should compare speech perception across people with normal hearing and hearing impairments using speech materials produced with and without wearing different mask types. This study did not calibrate sound level as its aim was to test within-subject factors; hence, real intensity level was deemed unnecessary. Future studies may measure real sound pressure levels in non-mask and mask conditions to further clarify the impact of mask-wearing on speech sound audibility. Findings presented were for standard surgical mask and KN95 mask only and may not be generalizable to other types of masks. In light of the use of cloth masks amongst members of the community, investigating the effect of fabric masks on both acoustic and auditory-perceptual measures is recommended.

## Conclusion

This study showed that the recorded acoustic voice signal changed whilst wearing either a standard surgical mask or a KN95 mask. Low/high spectral ratio increased i.e. spectral slope was steeper, which resulted from an attenuation of mean spectral levels in the 1–8 kHz regions relative to the 0–1 Hz regions. These spectral measures changed to a greater extent for KN95 mask than for surgical mask, suggesting that filtering and fitting characteristics of masks might determine the level of the voice and speech signal degradation. Alternatively, findings may reflect that the degree to which a speaker wearing a mask adjusts their phonation style is different across masks differing in characteristics. The findings appeared to imply that surgical masks might be a more relevant choice over the KN95 in COVID-19 pandemic to minimize the impact on communication.

HNR was improved in both surgical mask and KN95 conditions, implying possible filtering effects of these masks on spectral/glottal noise. This had implications in voice assessment in situations where patients are wearing a mask.

This study did not find significant changes in both CPPS and vocal intensity in the mask condition, implying that the design (e.g. recording setup) of the present study did not allow detection of changes in these measures, or that participants may have altered their vocal production in response to the mask.
